# Room-temperature *in situ* nuclear spin hyperpolarization from optically pumped nitrogen vacancy centres in diamond

**DOI:** 10.1038/ncomms9965

**Published:** 2015-12-07

**Authors:** Jonathan P. King, Keunhong Jeong, Christophoros C. Vassiliou, Chang S. Shin, Ralph H. Page, Claudia E. Avalos, Hai-Jing Wang, Alexander Pines

**Affiliations:** 1 Department of Chemistry, University of California, Berkeley, California 94720, USA; 2 Materials Sciences Division, Lawrence Berkeley National Laboratory, Berkeley, California 94720, USA

## Abstract

Low detection sensitivity stemming from the weak polarization of nuclear spins is a primary limitation of magnetic resonance spectroscopy and imaging. Methods have been developed to enhance nuclear spin polarization but they typically require high magnetic fields, cryogenic temperatures or sample transfer between magnets. Here we report bulk, room-temperature hyperpolarization of ^13^C nuclear spins observed via high-field magnetic resonance. The technique harnesses the high optically induced spin polarization of diamond nitrogen vacancy centres at room temperature in combination with dynamic nuclear polarization. We observe bulk nuclear spin polarization of 6%, an enhancement of ∼170,000 over thermal equilibrium. The signal of the hyperpolarized spins was detected *in situ* with a standard nuclear magnetic resonance probe without the need for sample shuttling or precise crystal orientation. Hyperpolarization via optical pumping/dynamic nuclear polarization should function at arbitrary magnetic fields enabling orders of magnitude sensitivity enhancement for nuclear magnetic resonance of solids and liquids under ambient conditions.

## Abstract (web summary)

Methods to increase nuclear spin polarization can enhance the sensitivity of magnetic resonance techniques however they are often limited to unfavourable conditions. Here, the authors achieve room temperature hyperpolarization of bulk ^13^C nuclear spins by exploiting the optical response of nitrogen vacancy centers in diamond crystals.

## Graphical abstract

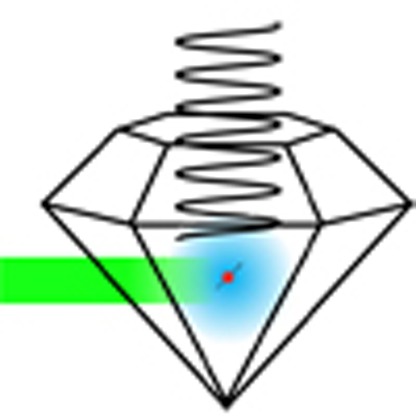

Nuclear magnetic resonance spectroscopy (NMR) and magnetic resonance imaging (MRI) are indispensable techniques in fields reaching from chemistry and materials to biology and medicine. Despite their non-destructive nature and broad range of applications they are subject to limited sensitivity. The sensitivity is primarily limited by the weak magnetization of nuclear spins, which is dependent on the population differences between nuclear spin states. At room temperature, the fractional excess of spin state population, or polarization, can be <1 p.p.m., motivating the development of methods to enhance NMR signals by generating polarization greater than prevails in thermal equilibrium. Such methods include: optical pumping applied to noble gases^1^,^2^,^3^,^4^ and semiconductors^5^,^6^; parahydrogen-induced polarization^7^,^8^,^9^; low temperature dynamic nuclear polarization (DNP)^10^,^11^,^12^,^13^; chemically induced DNP^14^; and optical pumping with DNP of excited triplet states in organic solids^15^. Despite the success of each of these techniques, they are limited to either low temperatures or specific molecules. Particularly desirable would be a general method to produce hyperpolarization at similar magnetic fields and temperatures as the NMR or MRI experiment. This enhancement would enable, for example, the observation of small quantities of biomolecules under biologically relevant conditions.

Recently, it has been recognized that nuclear spin hyperpolarization generated from nitrogen vacancy (NV^−^) centres in diamond could provide a platform for polarization transfer to NMR/MRI samples^16^,^17^,^18^,^19^. NV^−^ centres, with their optically polarized electron spin states and optical spin readout, have been the subject-of-interest for applications in quantum information, photonics and high-resolution sensing^20^, but here we are interested in their application to the polarization of nuclear spins. Nuclear spins hosted within the diamond lattice have been hyperpolarized using level anti-crossings that occur at specific crystal orientations and magnetic field strengths^17^,^18^. Evidence of nuclear spin hyperpolarization of proximate ^13^C spins was deduced from optically detected magnetic resonance (ODMR) spectra at the level anti-crossing fields and subsequently confirmed at a value of ∼0.5% in bulk by shuttling the diamond sample to a higher magnetic field for NMR detection^19^. These techniques were then extended to low fields away from the anti-crossing and to arbitrarily oriented NV^−^ centres using microwave irradiation^21^. Bulk ^13^C polarization has been generated at high field and low temperature and attributed to the coupling of the nuclear spins to the dipolar energy reservoir of the NV^−^ ensemble^16^, but the precise mechanism remains unclear. Furthermore, a recent proposal has suggested the use of shallow NV^−^ centres as a source for direct polarization transfer to a surrounding liquid^22^. In contrast, our method uses optical pumping of diamonds coupled with DNP under ambient conditions to obviate the need for cryogenic temperatures, sample shuttling, and precise crystal orientation and magnetic field strengths.

Here we show the generation of ^13^C nuclear spin polarization in diamond using microwave-driven DNP from the optically polarized electrons of NV^−^ defects. We demonstrate bulk nuclear spin polarization of 6%, an enhancement of ∼170,000 over thermal equilibrium. This is an order of magnitude greater than previous methods using NV^−^ centres^19^,^21^ and is achieved without precise control over magnetic field and crystal alignment. Room-temperature, hyperpolarized diamonds open the possibility of polarization transfer to arbitrary samples from an inert, non-toxic and easily separated source, a long sought-after goal of contemporary magnetic resonance.

## Results

### Optically pumped DNP

**Figure 1:**
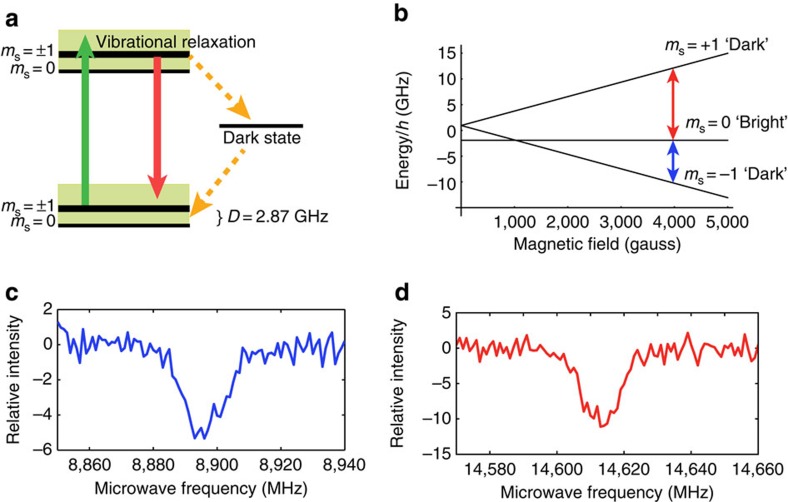
Optical pumping and optically detected magnetic resonance of nitrogen vacancy centres. (**a**) Energy levels and transitions for an NV^−^ centre in diamond. Optical pumping with green light at 532 nm induces transitions from the ground state spin-1 triplet to the excited triplet state. Subsequent to vibrational relaxation, fluorescence is detected in the red and near-IR. Spin conserving optical transitions and spin-dependent, non-radiative intersystem crossings lead to a preferential population of the *m*_s_=0 ground state, producing electron spin hyperpolarization of the NV^−^ centre. (**b**) Application of a magnetic field aligned along the NV^−^ axis lifts the degeneracy of the *m*_s_=±1 states, giving rise to two transitions that can be driven with microwave irradiation. The two transitions, (**c**) between *m*_s_=0 and *m*_s_=−1, and (**d**) between *m*_s_=0 and *m*_s_=+1, are observed by optically detected magnetic resonance (ODMR) through a reduction in the fluorescence intensity caused by a depletion of the ground *m*_s_=0 state. h=Planck's constant.

**Figure 2:**
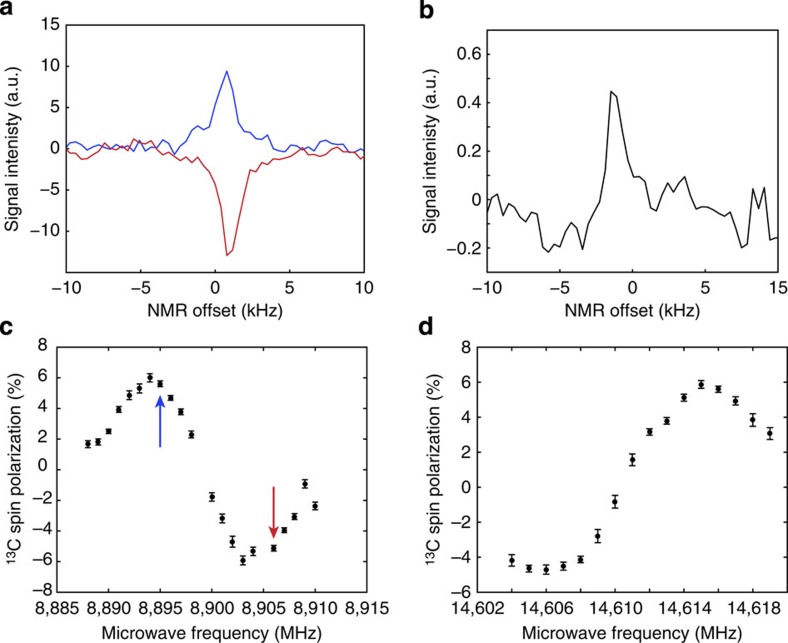
Hyperpolarization of nuclear spins. (**a**) ^13^C NMR spectra of natural abundance diamond after the accumulation of 60 scans under DNP for 60 s at 8,895 MHz (blue) and 8,907 MHz (red). (**b**) NMR spectrum of thermal equilibrium reference sample (99% ^13^C-enriched DMSO) after accumulating 12,676 scans. The diamond DNP signal corresponds to a polarization of 6%, an enhancement of ∼170,000 over thermal equilibrium. Consistent with known mechanisms of dynamic nuclear polarization, ^13^C nuclear polarization is an odd function of applied microwave frequency at the (**c**) *m*_s_=0 to *m*_s_=−1 and (**d**) *m*_s_=0 to *m*_s_=+1 NV^−^ transitions. The opposite signs of these two curves are consistent with the opposite electron spin polarizations of the two NV^−^ transitions. Data were acquired with a laser intensity of 16 W cm^−2^ and microwave power of 1.3 W. Error represents 95% confidence intervals for the amplitude of a Lorentzian fit to the frequency-domain data.

**Figure 3:**
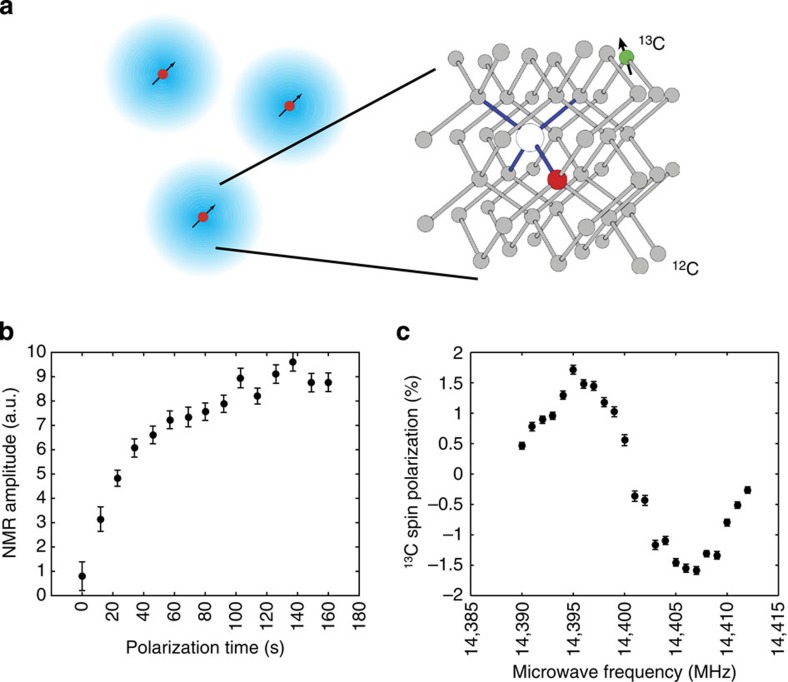
Dynamics and orientation dependence of hyperpolarization. (**a**) Schematic representation of the DNP process. Direct polarization near NV^−^ centres (red/white spheres in inset) gives rise to ^13^C (green spheres in inset) spin hyperpolarization. Spin diffusion carries the polarization (blue regions) from the NV^−^ centres (red circles) to the bulk material until a steady state is reached. (**b**) Time dependence of ^13^C spin polarization obtained by ^13^C NMR at 4.5 MHz. Beyond ∼100 s, the spin polarization has reached a steady state that represents a balance between the hyperpolarization/spin diffusion process and the spin-lattice relaxation of the nuclear spins. (**c**) Hyperpolarization achieved with NV^−^ centres misaligned 14° from the magnetic field. Error represents 95% confidence intervals for the amplitude of a Lorentzian fit to the frequency-domain data.

The NV^−^ centre in diamond comprises a substitutional nitrogen atom adjacent to a vacancy. Each NV^−^ centre has C_3*v*_ symmetry with the C_3_ axis aligned along one of the four equivalent [111] crystal axes. The electronic ground state of the negatively charged NV^−^ centre is a spin-1 triplet with a zero-field splitting *D*=2.87 GHz between the *m*_s_=±1 and *m*_s_=0 states (Fig. 1). The *m*_s_=0 state is preferentially populated via optical pumping with a 532 nm laser and spin-dependent non-radiative decay rates. Applying a magnetic field along the defect symmetry axis lifts the degeneracy of the *m*_s_=±1 states and gives rise to two distinct magnetic resonance transitions observable by ODMR. ODMR relies on a reduction in the fluorescence intensity induced by depopulation of the *m*_s_=0 state (Fig. 1a). In our experiment, ^13^C spins in a 4.5 mg diamond are hyperpolarized by DNP using an optically polarized microwave transition (red and blue arrows in Fig. 1b, spectra shown in Fig. 1c,d). DNP in diamond is known to occur via a combination of thermal mixing, where the dipolar energy reservoir of the interacting paramagnetic centres couples to the nuclei, and solid effect, where electron/nuclear spin flips are driven by microwave irradiation^23^ (See Supplementary Note 1 for a discussion of DNP mechanisms). The result of each of these mechanisms is the transfer of electron spin polarization to the nuclei. Owing to the optically polarized NV^−^ centres used for DNP, a strong, hyperpolarized NMR signal was observed from this natural isotopic abundance sample after accumulating 60 scans with a repetition time of 60 s (Fig. 2a). These parameters were chosen to achieve sufficient signal-to-noise in a reasonable experimental time and result in a polarization slightly lower than the maximum steady-state value relevant to a single scan. For comparison and calibration, after accumulating 12,676 scans with a repetition time of 10 ms, a 10 μl sample of liquid dimethyl sulfoxide (DMSO), enriched to 99% ^13^C and doped with Gd(III), produced an NMR signal of lower amplitude than the diamond by a factor of ∼12 (Fig. 2b). From the ratio of the numbers of ^13^C nuclei in the diamond and DMSO samples (0.015), the number of scans needed for each and the ratio of signal amplitudes, the maximum bulk ^13^C polarization in the diamond is estimated to be 6%. The odd function of polarization with respect to applied microwave frequency (Fig. 2c,d) is characteristic of DNP in solids, and the opposite signs of the signals in Fig. 2c,d are consistent with population of the *m*_s_=0 state of the NV^−^ centre. Since the width of the EPR transition is similar to the NMR frequency (∼4.5 MHz), it is expected that both solid effect and thermal mixing DNP mechanisms may be present^23^, and the frequency span between the maximum (occurring at 8,903 and 14,606 MHz) and minimum (8,894 and 14,615 MHz) polarizations is similar to, but not exactly, twice the NMR frequency. We attribute the dynamics to the coupled processes of DNP of nuclear spins proximate to the NV^−^ centres, nuclear spin diffusion to the bulk material^23^,^24^ and spin-lattice relaxation. This process is shown schematically in Fig. 3a and leads to a build-up of nuclear spin polarization over several minutes (Fig. 3b).

### Orientation dependence

The OP/DNP process is expected to be effective at arbitrary orientations of the NV^−^ defects, since the process depends on matching of the microwave frequency to a given transition, rather than a precise field strength and orientation. To test this idea, the sample was rotated 90° around the axis perpendicular to the laser and magnetic field. This ensured that no NV^−^ centres were aligned with the magnetic field. An ODMR signal was found at 14,402 MHz, which corresponds to an NV^−^ misalignment of 14° from the field. DNP data were collected at this frequency (Fig. 3c) showing ^13^C spin polarization approaching 2%. The reduction in DNP efficiency is attributed to the mixing of NV^−^ spin states and the corresponding reduction of polarization between the NV^−^ energy levels. However, high polarization should be present for all orientations except near the magic angle 
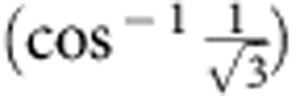
, where the states are an equal admixture of *m*_s_=±1 and *m*_s_=0. The effectiveness of the DNP for misaligned NV^−^ centres will be critical for the extension of this technique to randomly oriented powders, where polarization may be extracted from any crystal orientation via the integrated solid effect^15^. We further note that our technique should be general for a large range of magnetic fields. As long as the NV^−^ Zeeman interaction is significantly greater than the zero-field splitting, the physics presented here should be valid, including the robustness to orientation. Near to and below the level anti-crossing, the quantization axis will no longer be solely defined by the external field and this mechanism will no longer be valid, although other polarization mechanisms may exist^21^.

## Discussion

These results introduce a methodology for nuclear spin hyperpolarization in diamond that is robust to magnetic field strength and orientation. They demonstrate a bulk polarization of 6%, but with optimization of magnetic field, orientation and diamond samples the polarization could in theory approach the NV^−^ spin polarization, which is of order unity^20^. Hyperpolarized diamonds, which can be efficiently integrated into existing fabrication techniques to create high surface area diamond devices, including nanocrystal powders, will provide a general platform for polarization transfer. We envision highly enhanced NMR of liquids and solids using existing polarization transfer techniques, such as cross-polarization in solids^25^ and cross-relaxation in liquids^26^, or direct DNP to outside nuclei from shallow NV^−^ centres. Cross-relaxation has already been demonstrated as a method for polarization transfer between phases such as hyperpolarized ^129^Xe gas to solid^27^ and liquid^28^. These transfer techniques should be applicable to any sample that can be brought into intimate contact with the diamond surface, with the efficiency of polarization transfer determined by the relative rates of polarization transfer and spin-lattice relaxation. The efficiency of polarization transfer would approach 100% for long-T_1_ samples. Possible samples include liquids, solids and mildly frozen liquids (for example, glassy aqueous solutions) for solid-state OP/DNP with solid-state spin diffusion followed by thawing and observation of hyperpolarized liquid-state NMR. Hyperpolarization techniques based on optically polarized NV^−^ centres could enable enhanced sensitivity of magnetic resonance experiments, resulting in decreased experimental time and lower detection limits comparable to those of contemporary techniques such as dissolution DNP and chemical-specific, parahydrogen-induced polarization. NV^−^ centres as a polarization source could, however, be used at room temperature or under mild (near 0 °C) freeze/thaw conditions, which obviates the need for cryogenic instrumentation, shuttling between two high magnetic fields, and exogenous polarizing agents. The technique should be applicable to arbitrary target molecules, including biological systems that must be maintained at near ambient conditions.

## Methods

### Optically pumped DNP apparatus

To investigate DNP effects with NV^−^ centres, we constructed a combined DNP/optically detected magnetic resonance/NMR instrument, shown schematically in Supplementary Fig. 1. The magnetic field is supplied by a custom-built electromagnet (Tel-Atomic) and is set to 420 mT. A Coherent Verdi G15 laser delivers 532 nm illumination to the sample through a Gaussian beam with a waist of 1.5 mm, essentially illuminating the entire surface of the diamond with an intensity up to 16 W cm^−2^. The laser intensity was chosen to maximize polarization without excessive sample heating (see Supplementary Note 2 and Supplementary Fig. 2 for laser power dependence). Fluorescence is separated from excitation light by a dichroic mirror and detected by an avalanche photodiode. ODMR is performed by monitoring the diamond fluorescence while varying the applied microwave frequency. Microwave irradiation is delivered to the sample by a microwave loop of diameter 9.6 mm (See Supplementary Note 2 and Supplementary Fig. 3 for microwave power dependence). NMR was performed using a Magritek Kea 2 spectrometer with a homebuilt 50-turn planar coil probe tuned to ∼4.5 MHz.

### Diamond sample preparation

A commercially-available 2 mm × 2 mm × 0.32 mm, 〈100〉 surface-orientation single crystal of synthetic high-pressure, high-temperature diamond (Sumitomo) was acquired. Electron irradiation at 1 MeV with a fluence of 10^18^ cm^−2^ followed by annealing at 800 °C for 4 h in a mixture of 9% H_2_ and 91% He yielded an ensemble of NV^−^ centres. NV^−^ concentration is expected to be on the order of 10^18^ cm^−3^ under these conditions^29^. The crystal was mounted on a goniometer inside the electromagnet, and one of the 〈111〉 axes was aligned with the magnetic field by monitoring the ODMR spectrum. In this orientation, there are three equivalent ODMR spectra of the NV^−^ centres along 〈111〉 axes at an angle of 109.5° with respect to the magnetic field and a single ODMR spectrum corresponding to the aligned NV^−^ centres. With the field set to 420 mT, ODMR and DNP were performed using microwave fields at 8,900 and 14,600 MHz. For the misaligned NV^−^ data, the sample holder/NMR probe was rotated 90° around the vertical axis, which is perpendicular to both the magnetic field and the laser. A separate reference measurement (described later) was performed for this configuration in case of unintended variations of the NMR sensitivity.

### Experimental procedure

DNP data were acquired by polarizing for 60 s unless otherwise noted. Then, a 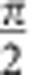 NMR pulse of duration 10 μs (calibrated via a nutation experiment) generated transverse magnetization that was inductively detected. Time-domain NMR data were apodized by exponential multiplication with a decay constant of 1 ms. After application of phase correction and a fast Fourier transform algorithm, frequency-domain spectra were fitted to single Lorentzian functions. The nuclear polarization was taken to be proportional to the amplitude of the fitted peak; error bars represent the 95% confidence intervals for the amplitude. The bulk polarization was calibrated relative to the thermal equilibrium signal of a 99% ^13^C-enriched sample of dimethyl sulfoxide, doped with gadolinium (III). The doping resulted in a spin-lattice relaxation time <2 ms, which allowed acquisition of ∼10,000 scans necessary for sufficient signal-to-noise ratio. See Supplementary Note 3 and Supplementary Fig. 4 for a discussion of the validity of the calibration. The polarization build-up was monitored with a saturation-recovery pulse sequence, in which the polarization was initially destroyed with a series of 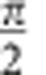 pulses followed by a variable polarization time and NMR detection. The build-up data were processed separately by exponential apodization and Fourier transform with Magritek Prospa software, followed by phase correction and fitting to Lorentzian functions to extract the amplitude.

## Additional information

**How to cite this article:** King, J. P. *et al*. Room-temperature *in situ* nuclear spin hyperpolarization from optically pumped nitrogen vacancy centres in diamond. *Nat. Commun.* 6:8965 doi: 10.1038/ncomms9965 (2015).

## Supplementary Material

Supplementary Information Supplementary Figures 1-4, Supplementary Notes 1-3 and Supplementary References.
