# Supplementary material for: Room-temperature in situ nuclear spin hyperpolarization from optically pumped nitrogen vacancy centres in diamond
Source: Nat Commun. 2015 Dec 7;6:8965. doi: 10.1038/ncomms9965 (PMC4686850; doi:10.1038/ncomms9965)
Supplement: Supplementary Information — Supplementary Figures 1-4, Supplementary Notes 1-3 and Supplementary References. [file ncomms9965-s1.pdf]

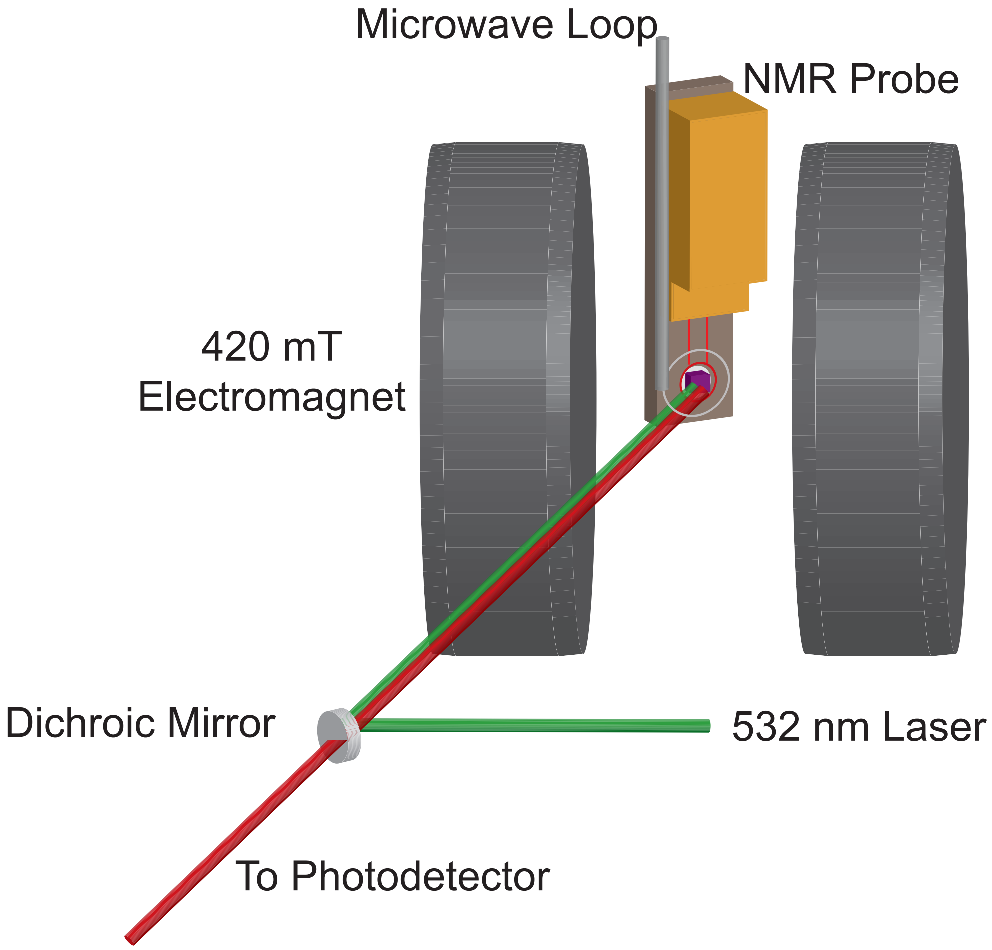

Supplementary Figure 1: **Experimental Setup for Optically Detected Magnetic Resonance, Dynamic Nuclear Polarization, and Nuclear Magnetic Resonance:** An unfocused 532 nm laser beam provides optical pumping and induces red/NIR sample fluorescence which is read out using a photodetector. A tuned NMR probe provides RF irradiation and detection while a microwave loop provides microwave irradiation.

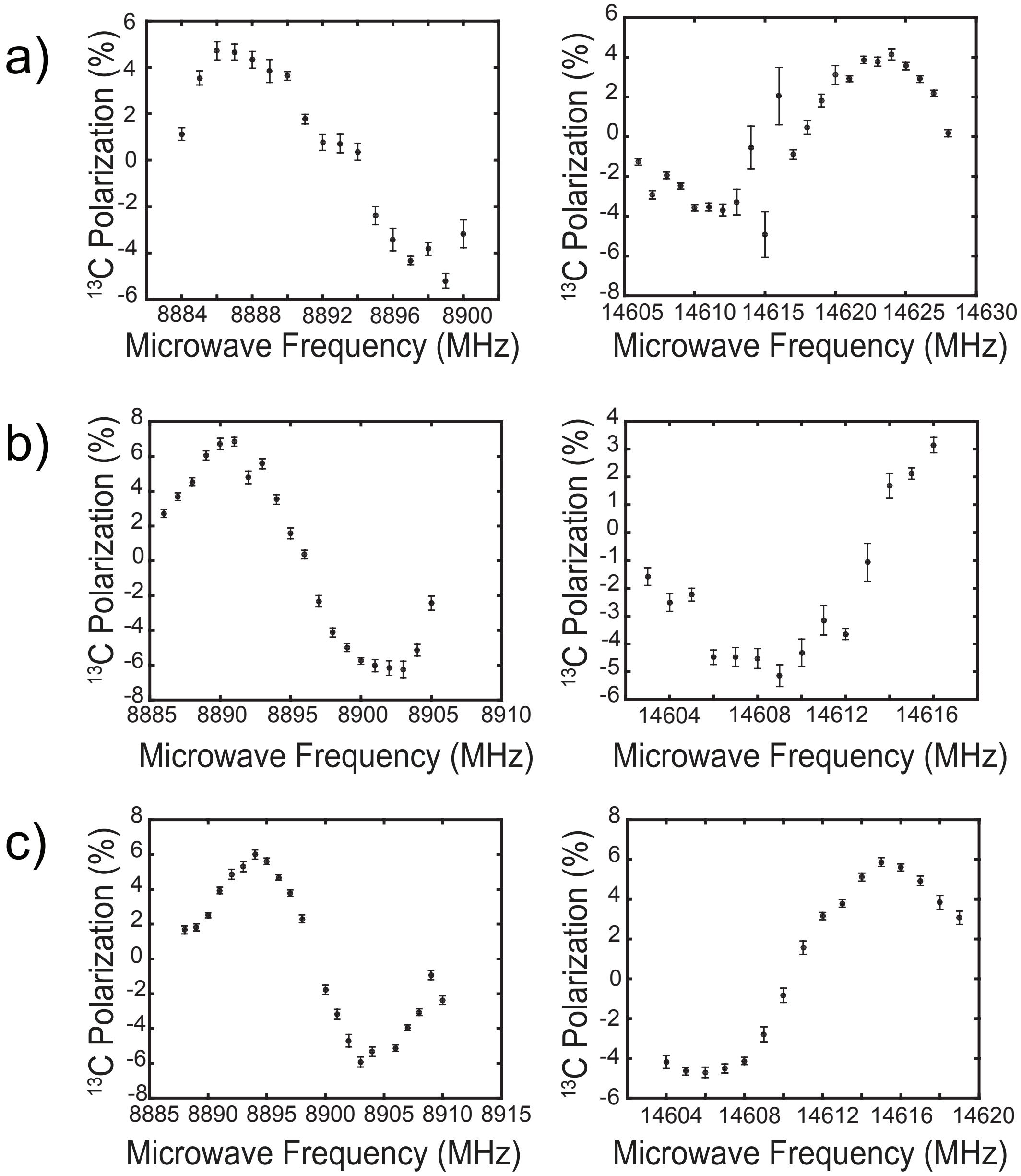

Supplementary Figure 2: **Hyperpolarization as a Function of 532 nm Laser Intensity:** a) 11 $\frac{\mathrm{W}}{\mathrm{cm}^2}$, b) 16 $\frac{\mathrm{W}}{\mathrm{cm}^2}$, c) 45 $\frac{\mathrm{W}}{\mathrm{cm}^2}$. Increased laser intensity results in a slight increase in nuclear polarization. The frequency shifts are attributed to the temperature dependence of the zero-field splitting

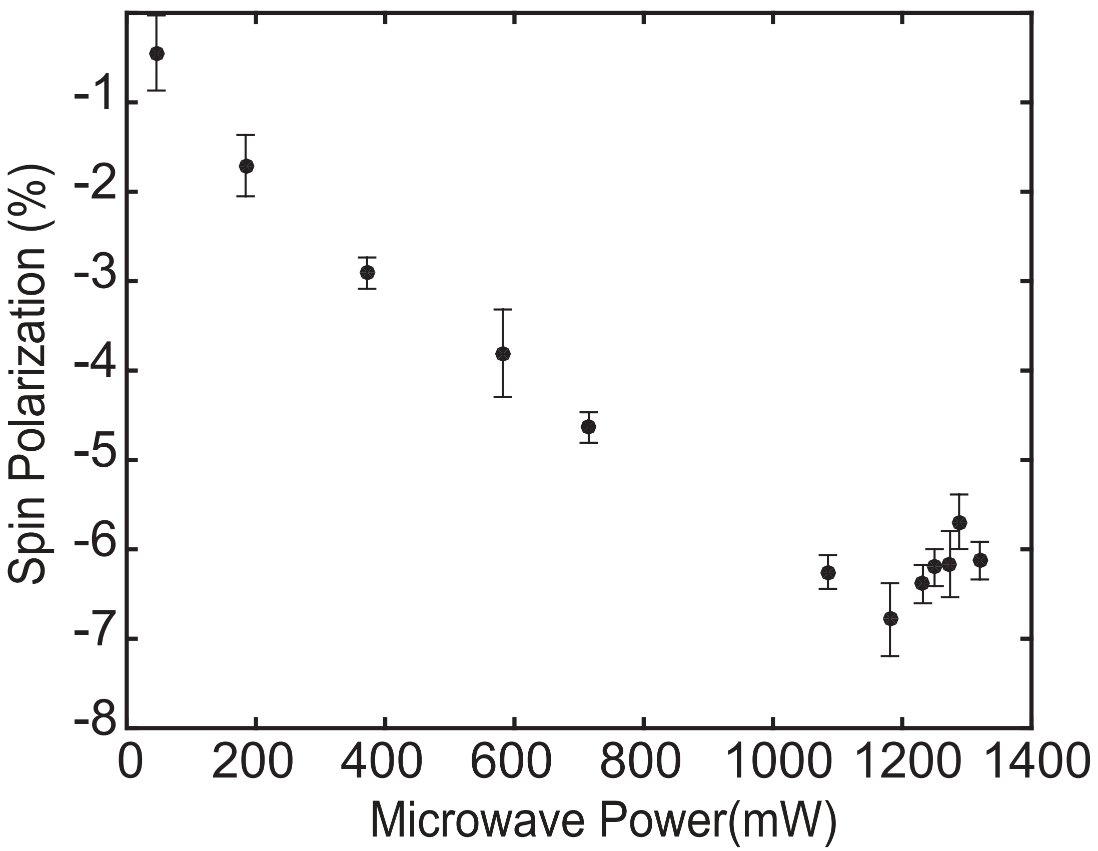

Supplementary Figure 3: **Hyperpolarization as a Function of Microwave Power:** Nuclear spin polarization as a function of microwave power after 60 s of DNP at 8896 MHz and 16 $\frac{W}{cm^2}$ illumination.

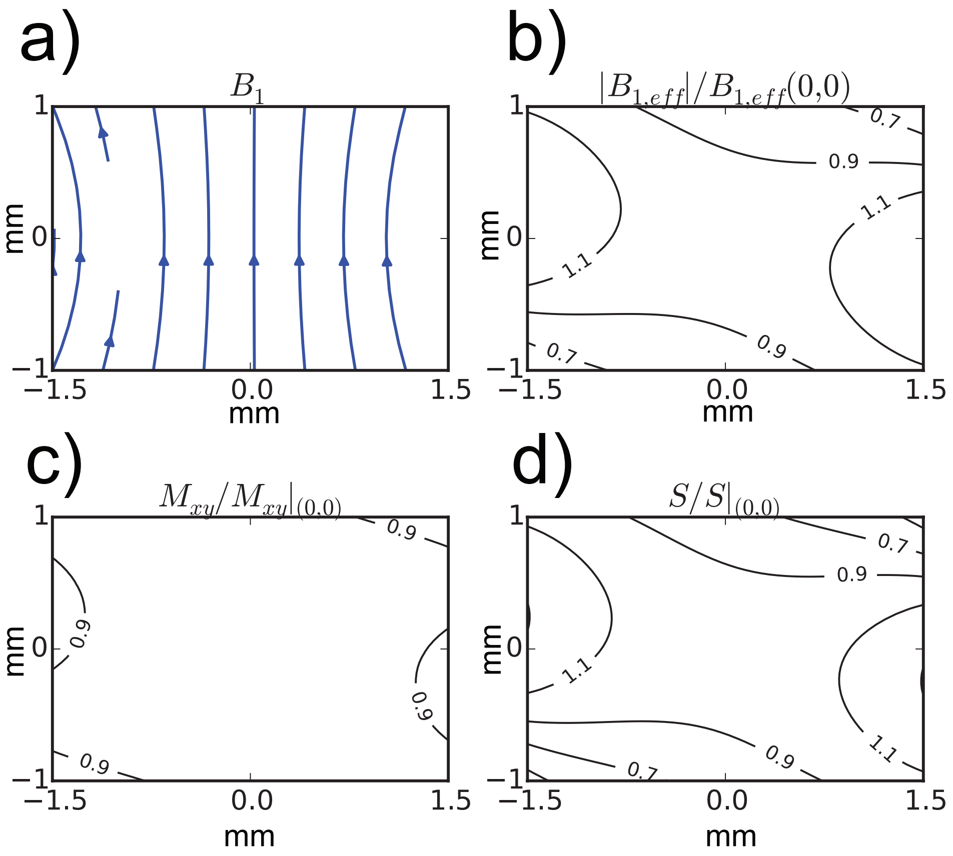

Supplementary Figure 4: $\mathbf{B}_1$ **Field Homogeneity:** a) Cross-section of field lines for RF pulses from planar NMR coil. b) Effective RF field perpendicular to the static magnetic field oriented 54.74° to the coil axis. c) Normalized nuclear magnetization generated by a nominal $\frac{\pi}{2}$ pulse. d) Overall NMR sensitivity as a function of position, accounting for generation of transverse nuclear magnetization and inductive sensitivity of the coil.

Supplementary Note 1

The mechanisms for DNP via fixed paramagnets in insulating solids are well known and described elsewhere (1; 2; 3). We briefly review the relevant mechanisms here. For a diamond containing ^{13}C and NV^{-} spins, there exist transitions at frequencies $\omega_{NV} \pm \omega_{^{13}C}$ involving simultaneous nuclear and electron spin flips that are nominally forbidden in the absence of electron-nuclear coupling. Dipolar coupling of these spins creates a non-zero transition probability, and a sufficiently strong microwave field may then drive the transition. These transitions are either zero quantum (induced by coupling terms of the form $S^{\pm}I^{\mp}$) or double quantum ($S^{\pm}I^{\pm}$) and may be selected by frequency. Since optical pumping of the NV^{-} center populates the $m_s = 0$ states, each of these transitions is polarized and the effect of driving the forbidden transitions is to preferentially induce nuclear spin flips of a particular sign. Thus, the net result of the combined optical pumping and microwave irradiation is to preferentially populate one of the nuclear spin states. This is known as the the solid effect method of DNP, since it relies on the dipolar interaction of fixed spins in solids (1; 2; 3).

If, however, the NV^{-} spin transitions are homogeneously broadened by their mutual dipole-dipole interactions, as is the case with high NV^{-} concentrations, then there exist energy-conserving transitions that involve a nuclear spin flip accompanied by multiple NV^{-} spin flips. In this case the dipolar energy reservoir of the NV^{-} centers is in thermal contact with the nuclear spins. This coupling can provide a relaxation pathway for the nuclear spins or, if the dipolar spin temperature is perturbed by optical pumping or microwave saturation, it can provide a second method of DNP known as thermal mixing (2; 4; 5). Application of microwave irradiation connects the dipolar

energy reservoir to the electron Zeeman energy in the rotating frame (6), and thus provides a pathway for electron spin polarization transfer to nuclei. The solid effect and thermal mixing DNP have similar dependencies on microwave frequency, where the maximum polarization occurs offset on either side of the ODMR transition. The results presented here likely contain contributions from both solid-state effect and thermal mixing effects. We expect the relative magnitudes of these effects to be sample dependent, owing to differing NV^- concentration.

Supplementary Note 2

The effectiveness of the OP/DNP process was found to increase slightly with laser intensity. While the exact relationship between laser intensity and NV^- spin polarization under these conditions is unknown, it is clear that optical absorption is significant over the depth of the diamond. The optical absorption coefficient in the diamond is approximately 9 mm^{-1}, and at thickness of 0.32 mm, only 6% of the light is transmitted. Increasing the laser light then effectively increases the volume of the sample that is highly polarized as well as possibly increasing the degree of polarization near the surface of the sample. Supplementary Figure 2 shows DNP data as a function of laser intensity. We also note the frequency shifts in the DNP data as a function of laser power; we attribute this to the temperature-dependence of the zero-field splitting (7).

The effectiveness of OP/DNP also increases with applied microwave power (Supplementary Figure 3). This is consistent with DNP mechanisms in diamond, where the solid effect or thermal mixing mechanisms of DNP are expected to contribute. In the case of the solid-effect, the increased

microwave power will more effectively drive the forbidden transitions involving mutual spin flips of ^{13}C and NV^{-} spins. For thermal mixing, the microwaves drive the NV^{-} dipole energy reservoir into equilibrium with rotating frame spin temperature of the NV^{-} centers. In each case, stronger microwave irradiation can result in more efficient transfer of polarization.

Supplementary Note 3

Typically, when performing quantitative NMR studies a reference sample of known quantity is mixed with the sample and observed under identical conditions. However, observing ^{13}C NMR at 420 mT is inherently difficult due to the low sensitivity, and without hyperpolarization it is impossible to observe an NMR signal with the same number of scans as the DNP experiments. We therefore used a 99% ^{13}C enriched sample of dimethyl sulfoxide, doped with gadolinium (III) to achieve a spin-lattice relaxation time less than 2 ms. This allowed accumulation of the more than 10,000 scans needed to achieve a sufficient signal-to-noise ratio for the thermally-polarized liquid whose polarization was 3.5×10^{-7}.

In order for the calibration to be accurate, several conditions must be met. First, all the parameters of the NMR experiment must remain constant, including pulse parameters and the quality factor of the resonant probe. Additionally, the reference sample should match the diamond in shape and be positioned identically with respect to the NMR coil to avoid miscalibration from the inhomogeneous sensitivity of the NMR coil. Care was taken to maintain all experimental parameters, but the problem of inhomogeneous sensitivity requires additional consideration. As it

was impractical to use a liquid sample with identical dimensions as the diamond (2 mm $\times$ 2 mm $\times$ 0.32 mm), a cylindrical liquid sample of diameter 3.7 mm and approximate depth 2 mm was used. Assuming the pulse parameters are calibrated to give a $\frac{\pi}{2}$ rotation at the center of the coil, the rotation angle was estimated as a function of position by numerically simulating the magnetic field (B_1) of the RF coil. Sensitivity to sample magnetization is also proportional to B_1, and these values were combined to create a map of sensitivity as function of position (Supplementary Figure 4). From these simulations it was estimated that the NMR sensitivity for the DMSO liquid reference sample was approximately 99% that of the diamond sample, and we conclude that RF field inhomogeneities do not cause significant error in the calibration.

There are a variety of other possible sources of error in the calibration of the diamond ^{13}C polarization, including the measurement of the number of spins in the diamond and DMSO sample, the deviation of the coil sensitivity from the numerical simulations, and possible drifts in the quality factor of the probe. However, the primary source of error is expected to be the noise relative to the weak calibration signal. This noise was characterized by fitting the DMSO spectrum with a Lorentzian function as described earlier and obtaining an error estimate of 6.7%. This error applies uniformly to the calibration of all DNP data and is thus not represented as individual error bars. This possible error does not affect the conclusions of this study.

Supplementary References

1. Slichter, C. P., *Principles of Magnetic Resonance.* New York: Springer, 3rd ed., (1996)

2. Reynhardt E., and High, G., "Dynamic nuclear polarization of diamond. I. Solid state and thermal mixing effects," *J. Chem. Phys.*, vol. 109, pp. 4090–4099, (1998)

3. Abragam, A., *Principles of Nuclear Magnetism.* Oxford: Oxford University Press, (1961)

4. Vanhouten, J., Wenckebach, W. T., and Poulis, N. J. "A study of the thermal contact between the nuclear zeeman system and the electron dipole-dipole interaction system," *Physica B and C*, vol. 92, no. 2, pp. 210–220, (1977)

5. King, J. P., Coles, P. J. and Reimer J. A., "Optical polarization of ^{13}C nuclei in diamond through nitrogen vacancy centers," *Phys. Rev. B.*, vol. 81, 073201, (2010)

6. Hovav, Y., Feintuch, A., and Vega, S. "Theoretical aspects of dynamic nuclear polarization in the solid state – spin temperature and thermal mixing," *Phys. Chem. Chem. Phys.*, vol. 15, no. 1, pp. 188–203, (2013)

7. Acosta, V. M., Bauch, E., Ledbetter, M. P., Waxman, A., Bouchard, L. -S., and Budker, D. "Temperature Dependence of the Nitrogen-Vacancy Magnetic Resonance in Diamond," *Phys. Rev. Lett.*, vol. 104, 070801, (2010)
